# ST8-t008-SCC*_mec_* IV methicillin-resistant *Staphylococcus aureus* in retail fresh cheese

**DOI:** 10.3168/jdsc.2023-0521

**Published:** 2024-04-20

**Authors:** Carolina Chaves, Melissa Montenegro, Hyrina Piedra, Marta Pérez-Villanueva, César Rodríguez

**Affiliations:** 1 Facultad de Microbiología, Universidad de Costa Rica, San Pedro de Montes de Oca, 11501-2060 San José, Costa Rica; 2 Centro de Investigaciones en Enfermedades Tropicales (CIET), Universidad de Costa Rica, San Pedro de Montes de Oca, 11501-2060 San José, Costa Rica

## Abstract

This study reports the finding of 3 ST8-t008-SCC*_mec_* IVa (2B) methicillin-resistant *Staphylococcus aureus* (MRSA) strains in fresh cheese purchased within a single market in Costa Rica. In line with the finding of the resistance genes *mecA*, *blaZ*, *mph*(C), and *msr*(A) in their genomes, these bacteria showed phenotypic resistance to multiple β-lactams and erythromycin. In addition, they carry genes for acquired resistance to aminoglycosides (*aph(3′)-III*) and fosfomycin (*fosD*), and genes for a myriad of virulence factors, including adhesins, hemolysins, and exotoxins. Our strains share multiple genomic features with MRSA from the USA300 lineage, which is a widely distributed and highly virulent strain implicated in community infections. As a result, consuming these or similar products could lead to multidrug infections in susceptible individuals. These results highlight safety deficiencies in cheese production practices and emphasize the risk of foodborne transmission of hard-to-treat ST8 MRSA strains.

## Highlights

• Three MRSA strains with multiple resistance genes were found in fresh cheese.
• These strains were classified as ST8-t008 and were shown to carry SCCmec IV cassettes.
• The strains display genomic signatures of USA300 MRSA, underscoring their clinical relevance.

## Graphical Abstract

**Figure:**
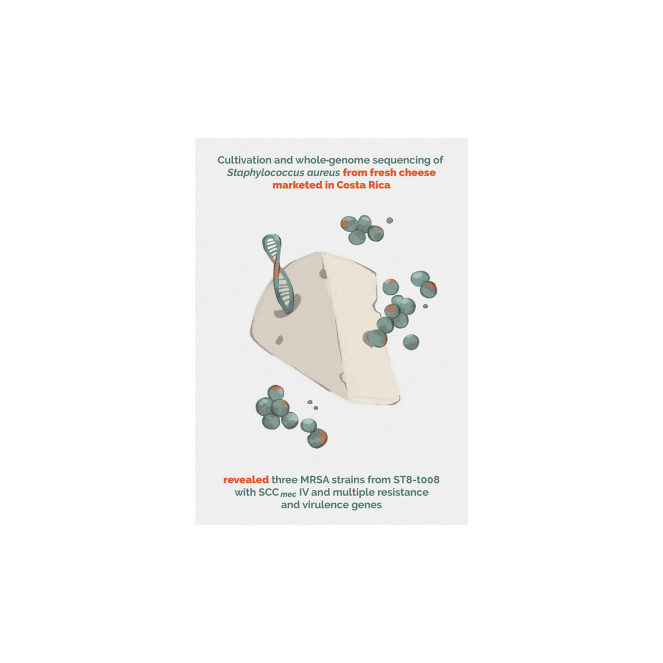
**Summary:** Three distinct ST8-t008-SCCmec IVa (2B) methicillin-resistant Staphylococcus aureus (MRSA) strains were identified in fresh cheese from a Costa Rican market. Exhibiting resistance to beta-lactams and erythromycin, these bacteria harbor additional genes, potentially conferring broader drug resistance. They also possess an array of toxin genes and share genomic markers with the clinically relevant USA300 Staphylococcus aureus lineage. These findings highlight the risk of foodborne ST8 MRSA transmission and underscore the need for enhanced safety measures in cheese production.

*Staphylococcus aureus*, a ubiquitous bacterium prevalent on human and animal skin and mucous membranes, includes virulent strains that can cause a broad spectrum of infections. Over time, this species has become a substantial global health concern for its implication in healthcare and community infections.

The rise of methicillin-resistant *S. aureus* (**MRSA**) has added complexity to *S. aureus* infection management. These unique strains are distinguished by the expression of *mecA* or *mecC* genes encoding altered penicillin-binding proteins with reduced β-lactam antibiotic affinity, which are included in a variety of staphylococcal cassette chromosome *mec* (**SCC*mec***) elements (da Silva Abreu et al., 2021; Naranjo-Lucena and Slowey, 2023).

Community transmission of *S. aureus* is predominantly through direct human interaction, animals, and animal-derived commodities (Brahma et al., 2022). This pathogen plays a role in contagious mastitis in dairy cattle and can be detected at multiple stages of the dairy production pipeline because dairy products provide favorable conditions for *S. aureus* growth and toxin synthesis (Gajewska et al., 2023).

Cheese is a globally consumed and popular food product. However, it has been implicated in foodborne illness outbreaks of various pathogens, including *S. aureus* (Rangel-Ortega et al., 2023). The presence of *S. aureus* in cheese is especially problematic due to its ability to produce heat-stable enterotoxins, which can lead to gastrointestinal symptoms in consumers.

This study aimed to investigate the presence and traits of MRSA in fresh cheese that was presumably prepared with pasteurized milk and marketed at Costa Rican retail stores. Through phenotypic testing and genomic analyses, we assessed their antibiotic resistance profiles and identified genes and genetic elements contributing to their resistance and virulence.

Isolates H1R2–1T, H1R3–4T, and H3R3–2T were obtained from fresh cheese samples collected on 2 separate occasions from 2 retailers in a single market in Costa Rica. These bacteria were cultivated by inoculating sample homogenates onto Baird-Parker agar plates and grew at population densities ranging from 10^1^ to 10^4^ cfu/g. Using the Vitek2 system (Bio-Mérieux), we confirmed that these isolates show phenotypic resistance to oxacillin (MIC >4 µg·mL^−1^) and other antibiotics (see below).

We implemented the random amplified polymorphic DNA (**RAPD**)-PCR procedure described by Reinoso et al. (2004) to evaluate the diversity of the isolates. These PCR reactions were prepared using a commercial Mastermix (ThermoScientific PCR Master Mix 2X), genomic DNA extracted with a commercial kit (NucleoSpin Tissue, Macherey-Nagel) from overnight cultures in trypticase soy broth, and oligonucleotides OLP6 (5′GAGGGAAGAG3′), OLP11 (5′ACGATGAGCC3′), and OLP13 (5′ACCGCCTGCT3′). The resulting amplicons were separated by electrophoresis on 1.5% agarose gels, which were prepared with 1× Tris-Borate-EDTA buffer (TBE) buffer.

The genomic DNA preparations presented above were also used for sequencing by synthesis at MicrobesNG (UK). In this regard, libraries were prepared using the Nextera XT Library Prep Kit (Illumina), and the sequencing was performed on a HiSeq platform (Illumina, 2x250 bp). Adapter and low-quality sequences were removed from the datasets using Trimmomatic 0.30, employing a sliding window quality cutoff of Q15. The resulting reads were then used for de novo assembly with SPAdes v3.7. Annotation of the assembled genomes was conducted using Prokka v1.13 (https://github.com/tseemann/prokka). The assembled genomes showed at least 30× coverage and N_50_ values ranging from 299 to 377 and 299 to 390 kb, respectively. Raw sequencing data can be downloaded from the following link: https://microbesng.com/portal/projects/8E75A3F4-F7B2-374B-8679-1EEEB9E3D896/.

The multilocus and SCC*mec* types of the isolates were determined with the MLST 2.0 (https://cge.food.dtu.dk/services/MLST/) and SCCmecFinder 1.2 (https://cge.food.dtu.dk/services/SCCmecFinder/) tools, respectively. Their spa types, instead, were assigned using the Ridom SpaServer (http://spaserver.ridom.de/). Parsnp (https://github.com/marbl/parsnp) was used to call pairwise SNPs using default settings. To identify the predicted resistome of the isolates, we used resfinder and the Resistance Gene Identifier (**RGI**) pipeline. In addition, we employed ABRicate (https://github.com/tseemann/abricate) in conjunction with the VFDB database (http://www.mgc.ac.cn/VFs/) to identify sequences encoding virulence factors.

All 3 isolates exhibited distinct RAPD patterns (Figure 1) and were distinguished from each other by 14 to 190 SNPs in their core genomes.

**Figure 1:**
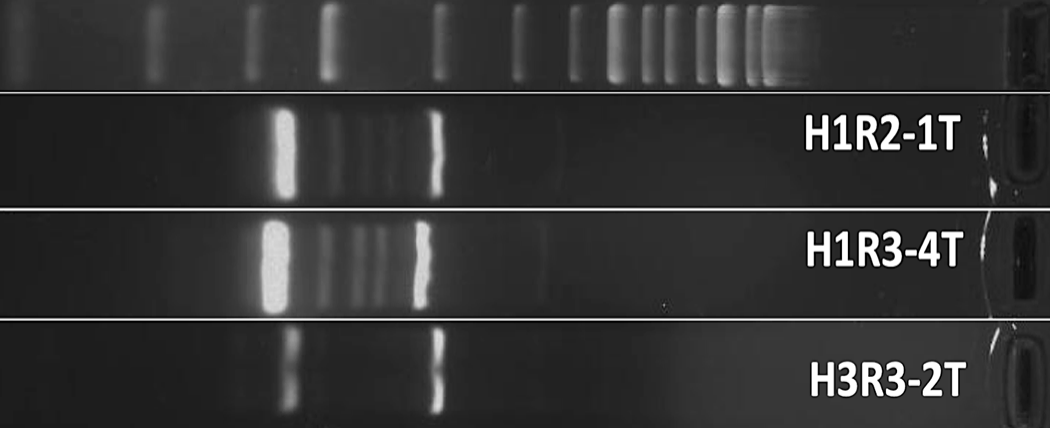
Random amplified polymorphic DNA (RAPD) patterns obtained for the 3 MRSA isolates analyzed.

Although they represent different strains, these bacteria were invariably assigned to the ST8-t008 lineage (Table 1). Furthermore, they were found to carry IVa-2B SCC*mec* elements (sequence similarity >90%; Table 1). All strains lack the arc and opp3 ACME clusters, PVL genes, and *seq* and *sek* genes from the pathogenicity island Pal5. Furthermore, only H1R2–1T and H1R3–4T possess a *mer*ARB operon, which confers resistance to mercury (Table 1).

**Table 1:** Genomic features of the MRSA isolates

Isolate	ST^1^	spa type	*SSC_mec_*	*copB*/*mco*	ACME (*arc* and *opp3* clusters)	PVL genes	PaI5 (*seq*, *sek*)	*gyrA*-84	*parC*-80	Plasmid-associated resistance genes^2^	*merABR*
H1R2–1T	8	t008	IVa-2B	ND^3^	ND	ND	ND	Ser^4^	Ser	Yes	Yes
H1R3–4T	8	t008	IVa-2B	ND	ND	ND	ND	Ser	Ser	Yes	Yes
H3R3–2T	8	t008	IVa-2B	ND	ND	ND	ND	Ser	Ser	Yes	ND

1 ST = sequence type.

2 Such as those found in p18805-p03.

3 ND = not detected.

4 Serine residue.

Our strains exhibited phenotypic resistance to a range of β-lactam antibiotics (penicillin, oxacillin, ampicillin, amoxicillin, and cephazolin) and erythromycin. By contrast, they were found to be susceptible to clindamycin, gentamicin, ciprofloxacin, linezolid, nitrofurantoin, tetracycline, trimethoprim-sulfamethoxazole, and vancomycin (Table 2). These findings were, for the most part, supported by our in silico analyses, which revealed hits with >98% identity and 100% coverage to *mecA* and *blaZ* for β-lactam resistance and *mph*(C) and *msr*(A) for macrolide resistance. Additionally, all 3 strains carried the *aph(3′)-III* gene (100% identity and coverage), conferring resistance to aminoglycosides, and *fosD* (79% identity and 100% coverage) associated with fosfomycin resistance. H1R2–1T and H1R3–4T were further distinguished by the presence of the tetracycline-resistance gene *tet*(K) (100% identity and coverage), whereas H1R2–1T also carried *erm*(C) gene (100% identity and coverage). In addition to these acquired antibiotic resistance genes, the 3 strains displayed hits to genes of the resistance plasmid p1885-p03 (Table 1), indicating the potential involvement of plasmids in the dissemination of antibiotic resistance among these MRSA strains.

**Table 2:** Antibiotic susceptibility testing^1^, ^2^

Strain	Beta-lactam					Macrolide- lincosamide		Aminoglycoside	Fluoroquinolone	Oxazolidinone	Nit	Tetracycline	Sxt	Glycopeptide
	Pen	Oxa	Amp	Amo	Cep	Ery	Cli	Gen	Cip	Lin		Tet		Van
H1R2–1T	R	R	R	R	R	R	S	S	S	S	S	S	S	S
H3R3–2T	R	R	R	R	R	R	S	S	S	S	S	S	S	S
H1R3–4T	R	R	R	R	R	I	S	S	S	S	S	S	S	S

1 Pen = penicillin; Oxa = oxacillin; Amp = ampicillin; Amo = amoxicillin; Cep = cefazolin; Ery = erythromycin; Cli = clindamycin; Gen = gentamicin; Cip = ciprofloxacin; Lin = linezolid; Nit = nitrofurantoin; Tet = tetracycline; Sxt = trimethoprim-sulfamethoxazole; Van = vancomycin.

2 R = resistant; S = susceptible; I = intermediate.

Several virulence factors were computationally detected in all 3 strains. These included genes encoding major autolysin (*atl*), clumping factor precursors (*cflAB*), extracellular adherence protein (*eap*), extracellular matrix-binding protein homolog (*ebh*), fibrinogen-binding protein (*efb*), intercellular adhesion locus (*icaABCD*), and hemolysin genes (*hla*, *hld*, *hlgABC*). Furthermore, toxin genes such as exfoliative toxin A (*eta*), leukocidin (*lukDE*), and a variety of exotoxins (*set30*, *set31*, *set34*, *set35*, *set36*, *set39*, *set40*) were also identified in these genomes.

Our study confirms that MRSA can be transmitted in the community through raw cheese consumption, posing a potential risk to consumers (Titouche et al., 2019). However, it should be noted that the magnitude of this risk is low, as indicated by our infrequent recovery of MRSA isolates despite extensive sampling efforts (3/211 isolates, data not shown) and recent literature (Schnitt and Tenhagen, 2020; Gajewska et al., 2023).

Although our phenotypic susceptibility testing did not include aminoglycosides and fosfomycin, our genomic analysis revealed the potential for multidrug resistance in the 3 strains. This worrisome trait, combined with the presence of genes encoding various virulence factors, further emphasizes the importance of our findings in terms of public health.

All 3 strains were classified as ST8-t008 and carried SCC*mec* IVa (2B) elements. This genomic profile was somewhat expected, as strains from clonal complex CC8, including ST8, have been previously detected in animal products (Herrera et al., 2016; Titouche et al., 2019). Additionally, MRSA strains carrying SCC*mec* IV have been shown to spread among livestock (Schnitt and Tenhagen, 2020).

In North and South America, strains belonging to the USA300 pulsotype are prevalent and notorious for their high virulence and enhanced environmental survival and transmission properties (Glaser et al., 2016). Our strains are t008 and ST8, display acquired plasmid-mediated resistance to clindamycin (*ermA* or *ermC*), tetracycline (*tetK* or *tetM*), and mupirocin (*mupA*), as well as chromosomal mutations conferring resistance to fluoroquinolones, possibly indicating their association with this pandemic lineage (Nimmo, 2012).

Variants of *S. aureus* USA300 and related strains can be differentiated based on the presence or absence of β-hemolysin converting phages, enterotoxin genes, and ACME and PVL genes. Our study detected such genetic variations in the 3 strains, highlighting the genomic diversity of MRSA and confirming that lateral gene transfer is a crucial driver of their evolution (Glaser et al., 2016).

This study reveals potentially highlights deficiencies in the manufacturing process or end product management. Accordingly, we advise retailers and the dairy industry to continuously review and strengthen their hygiene and disinfection measures.

We also advocate for surveillance programs focusing on raw materials, personnel, and finished products. A subsequent study analyzing clinical MRSA isolates from hospitals within the same geographical zones is also desirable, aiming to recognize potential niche overlaps. This could further reinforce the role of food in the proliferation of community-acquired MRSA.

In conclusion, the identification of multidrug, virulence factors, and genomic features of strains from the *S. aureus* USA300 lineage in our strains confirm their clinical relevance, underlining the importance of addressing food safety measures and implementing surveillance programs to mitigate the potential spread of community-acquired MRSA through food sources.
